# Prognostic value of cardiac magnetic resonance global T2 mapping in patients with takotsubo syndrome

**DOI:** 10.1093/ehjci/jeag243

**Published:** 2026-09-07

**Authors:** Yoshito Kadoya, Jerald Lim, Mohammed Alaqaili, Jamilah Mohammed Al-Harbi, Pape-Mamadou Sene, Mohammed El Mehdi El Yamani, Rebecca E Thornhill, Andrew M Crean, D Ian Paterson

**Affiliations:** Division of Cardiology, University of Ottawa Heart Institute, 40 Ruskin Street, Ottawa, ON K1Y 4W7, Canada; Department of Radiology, Radiation Oncology, and Medical Physics, University of Ottawa, Ottawa, ON, Canada; Division of Cardiology, University of Ottawa Heart Institute, 40 Ruskin Street, Ottawa, ON K1Y 4W7, Canada; Division of Cardiology, University of Ottawa Heart Institute, 40 Ruskin Street, Ottawa, ON K1Y 4W7, Canada; Division of Cardiology, University of Ottawa Heart Institute, 40 Ruskin Street, Ottawa, ON K1Y 4W7, Canada; Division of Cardiology, University of Ottawa Heart Institute, 40 Ruskin Street, Ottawa, ON K1Y 4W7, Canada; Department of Radiology, Radiation Oncology, and Medical Physics, University of Ottawa, Ottawa, ON, Canada; Division of Cardiology, University of Ottawa Heart Institute, 40 Ruskin Street, Ottawa, ON K1Y 4W7, Canada; Division of Cardiovascular Medicine, Faculty of Biology, Medicine and Health, University of Manchester, Manchester, UK; Division of Cardiology, University of Ottawa Heart Institute, 40 Ruskin Street, Ottawa, ON K1Y 4W7, Canada

## Abstract

**Aims:**

Myocardial oedema is a hallmark of takotsubo syndrome (TTS). Cardiac magnetic resonance (CMR) T2 mapping quantifies myocardial oedema, but its prognostic value in TTS is unclear. We evaluated the association between CMR-derived global myocardial T2 and major adverse cardiovascular and cerebrovascular events (MACE).

**Methods and results:**

We retrospectively identified consecutive TTS patients undergoing CMR with tissue mapping within 30 days of admission. Imaging was re-analysed, blinded to clinical data. The primary endpoint was MACE (all-cause death, heart failure hospitalization, recurrent TTS, stroke, or transient ischaemic attack). Time-dependent receiver operating characteristic (ROC) analysis derived a T2 threshold. Prespecified covariates were summarized by ridge-penalized Cox into a single linear predictor for adjustment. A Firth-penalized Cox model tested robustness. Among 110 patients (median age 68 years; 91% women), median global T2 was 56 ms (IQR 53–60), and 17 (15.5%) experienced MACE over a median follow-up of 763 days (IQR 525–1357). T2 ≥ 63 ms predicted 1-year MACE (AUC 0.671, sensitivity 56%, specificity 89%) and separated Kaplan–Meier curves (log-rank *P* = 0.003). In ridge-penalized Cox models, each 5 ms increase in global T2 was associated with MACE (HR 1.40, 95% CI 1.06–1.87; *P* = 0.020). This association was also observed after adjustment in the Firth-penalized model (HR 1.70, 95% CI 1.20–2.40; *P* = 0.004). An internally derived threshold of T2 ≥ 63 ms was also associated with MACE but showed modest 1-year discrimination (AUC 0.671).

**Conclusion:**

Global myocardial T2 may provide incremental prognostic information beyond prespecified clinical and imaging indices in TTS and refine risk stratification.

## Graphical Abstract

**Graphical Abstract:**
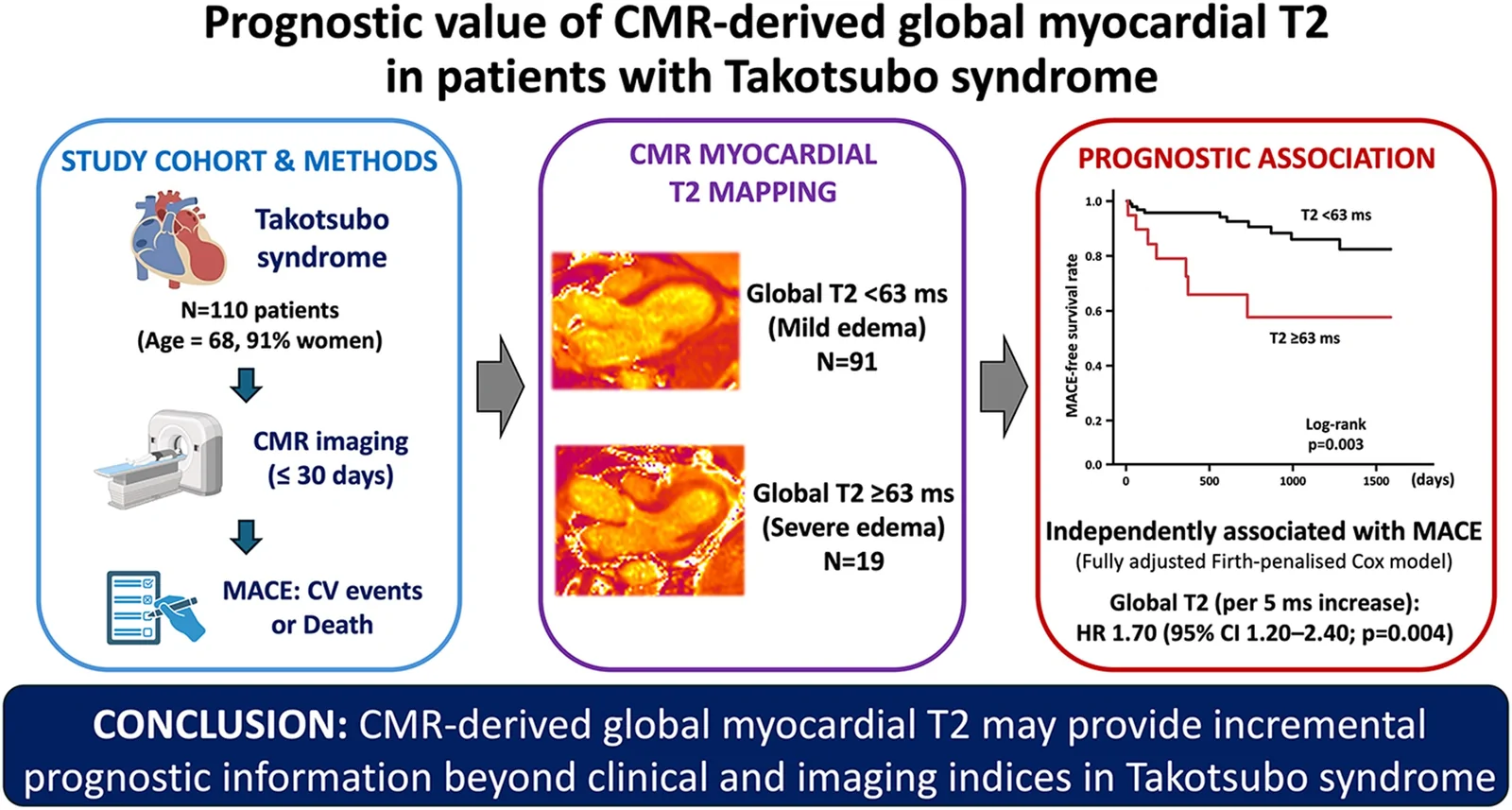

**See the editorial comment for this article ‘Myocardial oedema in Takotsubo syndrome: from diagnostic hallmark to prognostic marker?’, by M.-L. Luchian and M.Y. Desai, https://doi.org/10.1093/ehjci/jeag244.**

## Introduction

Takotsubo syndrome (TTS) is an acute cardiac condition characterized by transient left ventricular (LV) systolic dysfunction, typically precipitated by severe stressful events.^1,2^ Once considered a benign entity, TTS is now recognized to carry a substantial risk of adverse outcomes, including death, heart failure hospitalization, stroke, and recurrence, with morbidity and mortality rates comparable to those observed in acute coronary syndrome.^3,4^ While the underlying mechanisms remain incompletely defined, sympathetic nervous system overactivation and increased catecholamine secretion are thought to play a central role, leading to myocardial oedema and inflammation.^2,5–7^

Cardiac magnetic resonance (CMR) imaging is the gold-standard modality for assessing cardiac function and myocardial tissue characterization. Cardiac magnetic resonance is pivotal for establishing a definitive diagnosis and ensuring an appropriate differential diagnosis by distinguishing TTS from acute myocardial infarction or myocarditis.^8–12^ On CMR, TTS is identified by characteristic regional wall motion abnormalities, the presence of myocardial oedema, and the absence of ischaemic late gadolinium enhancement (LGE).^8–10^

Quantitative parametric mapping techniques, particularly T2 mapping, provide a validated, non-invasive and objective measure of myocardial water content, serving as a reliable indicator of myocardial oedema.^11^ Although previous studies have suggested an association between CMR mapping parameters and acute disease severity,^12,13^ it is unclear whether quantitative tissue mapping provides incremental prognostic information in patients with TTS.

Accordingly, we sought to evaluate whether myocardial T2 is associated with subsequent major adverse cardiovascular and cerebrovascular events (MACE) in patients with TTS and if it improves risk stratification beyond traditional indices.

## Methods

### Study population

We conducted a retrospective, single-centre study including consecutive patients with TTS who underwent clinically indicated CMR within 30 days of index admission between January 2020 and January 2025 (see Supplementary data online, *Figure S1*). The 30-day timeframe was chosen based on prior studies showing that LV dysfunction in TTS typically resolves within 4 weeks.^14,15^ TTS was defined by the International Takotsubo Diagnostic Criteria or the revised Mayo Clinic criteria.^1^ Patients without tissue mapping or LGE images, or with image quality precluding analysis, were excluded. Clinical and demographic data were abstracted from electronic medical records (Epic Systems, WI, USA). Obstructive coronary artery disease (CAD) was defined as ≥70% stenosis on invasive coronary angiography or coronary computed tomography angiography. The study complied with the Declaration of Helsinki (2013); institutional review board approval was obtained (20240471-01H) with a waiver of written informed consent owing to the retrospective design.

### CMR acquisition and image analysis

CMR was performed on a 1.5 T scanner (Magnetom Aera; Siemens Healthineers, Erlangen, Germany) using a standard protocol,^16^ beginning with cine imaging in long-axis (2-, 3-, 4-chamber) planes and a contiguous short-axis stack using a balanced steady-state free precession sequence with 8 mm slice thickness. Native (5(3)3) and post-contrast (4(1)3(1)2) modified Look-Locker inversion recovery T1 maps were acquired in three 8 mm-thick short-axis oblique views (basal, mid-ventricular, and apical) and in the 3- and 4-chamber views. Using the same slice prescriptions, T2 maps were acquired using T2-prepared steady-state free precession images with three T2-preparation times (0, 25, and 55 ms). In-line image registration and motion correction were applied for both T1 and T2 maps (Siemens Healthineers, Erlangen, Germany). LGE imaging was performed 8–10 min after administering 0.15 mmol/kg of a gadolinium-based contrast agent (gadobutrol, Bayer Healthcare, Berlin, Germany) using free-breathing segmented gradient echo with phase-sensitive inversion recovery and respiratory motion-corrected averaging. Late gadolinium-enhanced images were acquired in long-axis (2-, 3-, 4-chamber) planes and contiguous short-axis stack.

Images were re-analysed (cvi42 version 6.0; Circle Cardiovascular Imaging, Calgary, Canada) by two level-3 cardiologists with at least 3 years of CMR experience each, blinded to clinical data. Regional LV wall motion was categorized visually as normal, hypokinetic, akinetic, or dyskinetic across basal, mid, and apical zones according to the worst segment within each region. For quantitative analysis of cardiac function and myocardial tissue mapping, the endocardial and epicardial LV borders were traced semi-automatically and manually corrected; the papillary muscles and trabeculations were included in the LV volume. Regions with significant artefact on tissue mapping and LGE images were excluded from analysis. LGE was assessed visually as ischaemic or non-ischaemic according to the pattern of myocardial involvement. Myocardial extracellular volume (ECV) was derived from pre-contrast and post-contrast T1 times, as well as haematocrit value, calculated using the formula ECV = (1 - haematocrit) × (ΔR1myocardium/ΔR1blood).

Global T2 was defined as the mean value from all three short-axis slices (basal, mid-ventricular, and apical) after artefact exclusion. Segmental T2 was reported as the mean within each of the three slices. A region-guided T2 was additionally defined as the mean of the slice(s) with the worst wall-motion grade per patient. Maximal segmental T2 was additionally defined as the slice (basal, mid-ventricular, or apical) with the highest mean T2, irrespective of wall-motion abnormality. Identical computations were applied to native T1 and ECV. Institutional reference ranges (derived from healthy volunteer scans acquired with an identical 1.5 T protocol) were global T2 < 55 ms and native T1 < 1040 ms.

### Clinical endpoint

The primary endpoint was MACE, defined as a composite of all-cause death, heart failure hospitalization, recurrent TTS, and stroke or transient ischaemic attack. Endpoints were defined as follows: all-cause death (any documented death) and heart failure hospitalization (unplanned admission with congestion and intensification of diuretic or vasoactive therapy); recurrent TTS, defined as a new episode meeting the InterTAK or revised Mayo Clinic diagnostic criteria >30 days after the index event; and stroke (focal neurological deficit with corroborating imaging) and transient ischaemic attack (focal deficit <24 h without imaging correlate). Outcomes were ascertained by a comprehensive review of electronic medical records. For each patient, the first qualifying event was counted; patients without events were censored at the last clinical contact.

### Statistical analysis

Categorical variables were summarized as counts (percentages) and continuous variables as mean ± SD or median (interquartile range), according to the distribution assessed with the Shapiro–Wilk test. Group comparisons used Pearson’s χ^2^ test or Fisher’s exact tests for categorical variables and Student’s *t*-test or Mann–Whitney *U* test for continuous variables, as appropriate. To evaluate the regional differences of T1, T2, and ECV values across myocardial segments (basal, mid, apical), Friedman’s test or repeated-measures analysis of variance was used as appropriate.

Discrimination of global T2 for 1-year MACE was assessed using time-dependent receiver operating characteristic (ROC) analysis, reporting the 1-year area under the curve (AUC) and the threshold that maximized the Youden index. Time-dependent AUCs were additionally estimated at 2 and 3 years to capture medium-term discrimination. To quantify the optimism of the data-driven threshold and the model C-index, we performed 1000 bootstrap resamples with re-selection of the Youden-optimal threshold and re-fitting of the Cox model on each replicate. Kaplan–Meier curves were plotted for the derived T2 threshold and compared with the log-rank test.

To mitigate overfitting given the limited number of MACE events, we used a prespecified ridge-penalized Cox modelling strategy to construct an adjustment score from baseline covariates. Specifically, we fitted ridge-penalized Cox models using (i) a clinical covariate set (age, sex, atrial fibrillation, neurologic trigger, obstructive CAD ≥70%, and atypical TTS phenotype) and (ii) a combined clinical + imaging covariate set that additionally included LV ejection fraction (EF), right ventricular (RV) EF, presence of ischaemic LGE, and LV mass index.^4,17,18^ The ridge penalty (λ) was selected by 10-fold cross-validation to minimize the cross-validated partial log-likelihood deviance. For each patient, we then computed the model-based linear predictor, which served as a single composite adjustment variable (1 degree of freedom), summarizing the prespecified covariates.

We then evaluated the incremental prognostic value of global myocardial T2 by adding T2 to Cox models that included the ridge-derived adjustment score; the same model framework was used for the sensitivity analyses. The fully adjusted Firth-penalized Cox model with all covariates entered individually provided a complementary robustness check, and Firth penalization was also applied as a fallback estimator in per-component models with very small event counts. T2 was modelled primarily as a continuous exposure (per 5 ms increase) and secondarily as a binary indicator (T2 ≥ 63 ms) derived from the time-dependent ROC analysis. Improvement in model fit with the addition of T2 was assessed by the likelihood-ratio test (LRT) and Akaike Information Criterion (AIC). Discrimination was quantified using Harrell’s C-index, and calibration was assessed with a bootstrap-corrected calibration plot at 1 year (m = 25 per group, B = 200 resamples). The functional form of continuous T2 was tested by comparing a 3-knot restricted cubic spline with a linear model using the likelihood-ratio test and AIC. The proportional hazards assumption was examined using Schoenfeld residuals.

Sensitivity analyses comprised (i) segmental (basal, mid, apical), maximal segmental, and region-guided T2 (mean T2 of the slice with the worst wall-motion grade) entered as alternative exposures in ridge-adjusted Cox models; (ii) stratification and interaction analyses for the admission-to-CMR interval; (iii) per-component analyses for each element of the composite endpoint; (iv) further adjustment for transthoracic echocardiography (TTE) parameters [LVEF, LV outflow tract (LVOT) obstruction, moderate-or-greater mitral regurgitation, RV dysfunction] and LVEF recovery from the baseline TTE at admission to follow-up TTE or CMR in the subset with complete admission TTE data; (v) further adjustment for in-hospital therapy [beta-blocker, angiotensin-converting enzyme inhibitor (ACE-I)/angiotensin receptor blockers (ARB), diuretic, inotrope; mechanical circulatory support was not used in any patient and was therefore excluded from the adjustment set]; and (vi) exclusion of patients with ischaemic LGE. As a sub-analysis, native myocardial T1 replaced myocardial T2 in the ridge-based and Firth-penalized models to allow direct comparison between the two parameters (native T1 modelled per 100 ms increase and as a binary variable T1 ≥ 1237 ms, derived from the time-dependent ROC analysis).

All analyses used two-sided testing with α=0.05 and were performed in R version 4.2.2 (R Foundation for Statistical Computing).

## Results

### Patient characteristics

Among 127 screened patients, 110 were included (median age 68 years; 91% women) for the final analysis (*Table 1*). A history of psychiatric disorder was identified in 43 (39.1%), and 48 reported an emotional trigger (43.6%) for their event. A typical TTS phenotype occurred in 74 (67.3%), and 36 (32.7%) were atypical, including mid-ventricular in 27 (24.5%), reverse in 4 (3.6%), and focal in 5 (4.5%). Atrial fibrillation was more frequent among patients who experienced MACE than those who did not (29.4% vs. 6.5%, *P* = 0.013). TTE data on admission were available for 106 patients (96.4%): mean LVEF 42 ± 12%, LVOT obstruction in 8 (7.5%), moderate-or-greater mitral regurgitation in 3 (2.8%), and RV dysfunction in 14 (13.2%). In-hospital medical therapy was retrievable in all 110 patients: beta-blockers in 84 (76.4%), ACE-I/ARBs in 95 (86.4%), diuretics in 21 (19.1%), and inotrope in 15 (13.6%); no patient required mechanical circulatory support (0/110). In-hospital inotropic support was more frequent among patients who experienced MACE (35.3% vs. 9.7%, *P* = 0.012). Documented LVEF recovery from the baseline TTE at admission to follow-up TTE or CMR was observed in 103/106 (97.2%).

**Table 1:** Patient characteristics in the overall cohort and in subgroups stratified according to clinical events

	Total (*n* = 110)	MACE (+) (*n* = 17)	MACE (−) (*n* = 93)	*P*-value
**Baseline characteristics**				
**Demographics**				
Age, years	68 (59 to 74)	74 (61 to 79)	66 (59 to 73)	0.059
Female, *n* (%)	100 (90.9)	16 (94.1)	84 (90.3)	1.000
Body mass index, kg/m^2^	25.1 (21.5 to 29.3)	25.0 (21.7 to 27.3)	25.2 (21.5 to 29.4)	0.846
Body surface area, m^2^	1.76 (1.57 to 1.88)	1.77 (1.60 to 1.84)	1.75 (1.55 to 1.91)	0.928
**Cardiovascular risk factors**				
Hypertension, *n* (%)	68 (61.8)	10 (58.8)	58 (62.4)	0.792
Dyslipidaemia, *n* (%)	50 (45.5)	7 (41.2)	43 (46.2)	0.794
Diabetes mellitus, *n* (%)	18 (16.4)	0 (0.0)	18 (19.4)	0.069
Current smoking, *n* (%)	20 (18.2)	3 (17.6)	17 (18.3)	1.000
Family history of CAD, *n* (%)	24 (21.8)	1 (5.9)	23 (24.7)	0.113
**Past medical history**				
Prior PCI, *n* (%)	8 (7.3)	3 (17.6)	5 (5.4)	0.105
Prior CABG, *n* (%)	1 (0.9)	0 (0.0)	1 (1.1)	1.000
Prior MI, *n* (%)	4 (3.6)	2 (11.8)	2 (2.2)	0.112
Prior TTS, *n* (%)	8 (7.3)	2 (11.8)	6 (6.5)	0.607
Psychiatric disorder, *n* (%)	43 (39.1)	10 (58.8)	33 (35.5)	0.103
Atrial fibrillation, *n* (%)	11 (10.0)	5 (29.4)	6 (6.5)	0.013
Paroxysmal	7 (6.4)	3 (17.6)	4 (4.3)	0.073
Long-standing/persistent	4 (3.6)	2 (11.8)	2 (2.2)	0.112
**Symptoms**				
Chest pain, *n* (%)	76 (69.1)	9 (52.9)	67 (72.0)	0.154
Dyspnoea, *n* (%)	18 (16.4)	5 (29.4)	13 (14.0)	0.150
Syncope, *n* (%)	8 (7.3)	1 (5.9)	7 (7.5)	1.000
Others, *n* (%)	8 (7.3)	2 (11.8)	6 (6.5)	0.607
**Triggers**				
Emotional, *n* (%)	48 (43.6)	8 (47.1)	40 (43.0)	0.795
Physical, *n* (%)	41 (37.3)	6 (35.3)	35 (37.6)	1.000
Neurologic, *n* (%)	4 (3.6)	2 (11.8)	2 (2.2)	0.112
Undetected, *n* (%)	17 (15.5)	1 (5.9)	16 (17.2)	0.464
**Medications**				
Aspirin, *n* (%)	19 (17.3)	3 (17.6)	16 (17.2)	1.000
ACE-I/ARB, *n* (%)	39 (35.5)	9 (52.9)	30 (32.3)	0.166
Beta-blocker, *n* (%)	23 (20.9)	8 (47.1)	15 (16.1)	0.008
Calcium-blocker, *n* (%)	19 (17.3)	1 (5.9)	18 (19.4)	0.296
Statin, *n* (%)	44 (40.0)	11 (64.7)	33 (35.5)	0.032
Diuretics, *n* (%)	11 (10.0)	3 (17.6)	8 (8.6)	0.371
Anticoagulation, *n* (%)	13 (11.8)	5 (29.4)	8 (8.6)	0.029
**TTS phenotype**				
Typical (Apical), *n* (%)	74 (67.3)	12 (70.6)	62 (66.7)	0.972
Atypical	36 (32.7)	5 (29.4)	31 (33.3)	0.972
Mid-ventricular, *n* (%)	27 (24.5)	3 (17.6)	24 (25.8)	0.557
Reverse, *n* (%)	4 (3.6)	1 (5.9)	3 (3.2)	0.494
Focal, *n* (%)	5 (4.5)	1 (5.9)	4 (4.3)	0.575
**Laboratory findings**				
White blood cell, ×10^9^/L	10.2 (8.0 to 14.2)	10.6 (9.2 to 13.0)	10.1 (7.6 to 14.2)	0.315
Haemoglobin, g/L	133 (122 to 144)	127 (116 to 140)	134 (124 to 145)	0.237
Creatinine, umol/L	65 (53 to 85)	70 (53 to 92)	64 (53 to 85)	0.498
Troponin T, ng/L^a^	501 (193 to 947)	489 (118 to 995)	519 (198 to 947)	0.836
**ECG findings**				
ST elevation, *n* (%)	39 (35.5)	7 (41.2)	32 (34.4)	0.593
ST depression, *n* (%)	18 (16.4)	4 (23.5)	14 (15.1)	0.474
T inversion, *n* (%)	37 (33.6)	5 (29.4)	32 (34.4)	0.786
LBBB, *n* (%)	7 (6.4)	1 (5.9)	6 (6.5)	1.000
**TTE findings (*n* = 106)** ^b^				
LVEF, %	42 ± 12	42 ± 15	42 ± 12	0.942
Wall motion score index	1.94 (1.63 to 2.18)	1.75 (1.56 to 2.06)	1.94 (1.63 to 2.19)	0.299
LVOT obstruction (PG ≥30 mmHg), *n* (%)	8 (7.5)	0 (0.0)	8 (9.0)	0.350
Moderate-or-greater MR, *n* (%)	3 (2.8)	0 (0.0)	3 (3.4)	1.000
RV dysfunction, *n* (%)	14 (13.2)	3 (17.6)	11 (12.4)	0.695
**Coronary angiography findings**				
Obstructive CAD, *n* (%)	20 (18.2)	4 (23.5)	16 (17.2)	0.508
**In-hospital therapy**				
Medications				
Beta-blocker, *n* (%)	84 (76.4)	14 (82.4)	70 (75.3)	0.758
ACE-I/ARB, *n* (%)	95 (86.4)	15 (88.2)	80 (86.0)	1.000
Diuretics, *n* (%)	21 (19.1)	3 (17.6)	18 (19.4)	1.000
Inotropic support, *n* (%)	15 (13.6)	6 (35.3)	9 (9.7)	0.012
Mechanical circulatory support, *n* (%)	0 (0.0)	0 (0.0)	0 (0.0)	1.000
Documented LVEF recovery, *n* (%)^c^	103 (97.2)	16 (94.1)	87 (97.8)	0.411

^a^Peak value during admission.

^b^Baseline TTE data were retrievable in 106 of 110 patients (96.4%); the remaining 4 had echocardiography performed at an outside institution with non-retrievable image archives or had image quality that precluded analysis.

^c^LVEF recovery is defined as a normalization of LVEF to ≥50% from baseline TTE at admission to follow-up TTE/CMR (binary indicator). Percentages are calculated within patients with available admission TTE (overall *n* = 106; MACE + *n* = 17, MACE− *n* = 89).

ACE-I, angiotensin-converting enzyme inhibitor; ARB, angiotensin receptor blockers; CABG, coronary artery bypass grafting; CAD, coronary artery disease; ECG, electrocardiogram; LBBB, left bundle branch block; LVEF, left ventricular ejection fraction; MACE, major adverse cardiovascular and cerebrovascular events; MI, myocardial infarction; PCI, percutaneous coronary intervention; PG, peak gradient; TTE, transthoracic echocardiography; TTS, takotsubo syndrome.

### CMR findings

The median time from admission to CMR was 4 days [interquartile range (IQR) 2–6] (*Table 2*); 70.9% of scans were within 5 days and 90.0% within 10 days (see Supplementary data online, *Figure S4*). Mean LVEF was 47 ± 12%, and the median RVEF was 58% (IQR 52–63). Global native myocardial T1 was 1102 ± 71 ms, global ECV 32% (IQR 28–34) and global myocardial T2 56 ms (IQR 53–60); no segments were excluded from the tissue mapping analysis (see Supplementary data online, *Figure S2* for case example). Segmental tissue mapping showed a significant regional gradient for native T1, T2 and ECV, with the lowest values in the basal segments (*P* < 0.01 for each); T2 and ECV rose progressively towards the apex, whereas native T1 peaked at the mid-ventricular level.

**Table 2:** CMR findings in the overall cohort and in subgroups stratified according to clinical events

Parameters	Total (*n* = 110)	MACE (+) (*n* = 17)	MACE (−) (*n* = 93)	*P*-value
**Admission to CMR interval, days**	4 (2 to 6)	4 (2 to 5)	4 (2 to 6)	0.910
**Biventricular size and function**				
LVEDV index, mL/m^2^	74 ± 17	76 ± 19	74 ± 17	0.658
LVESV index, mL/m^2^	39 (28 to 49)	41 (31 to 50)	38 (28 to 49)	0.469
LV mass index, g/m^2^	52 (46 to 57)	47 (44 to 53)	52 (46 to 59)	0.059
LVEF, %	47 ± 12	46 ± 11	47 ± 13	0.657
RVEDV index, mL/m^2^	60 ± 15	61 ± 20	60 ± 14	0.783
RVESV index, mL/m^2^	25 (19 to 31)	26 (21 to 30)	25 (19 to 31)	0.694
RVEF, %	58 (52 to 63)	55 (48 to 63)	58 (52 to 63)	0.588
**LV wall motion**				
**Basal segment**				0.029
Normal, *n* (%)	97 (88.2)	12 (70.6)	85 (91.4)	
Hypokinesis, *n* (%)	12 (10.9)	5 (29.4)	7 (7.5)	
Akinesis, *n* (%)	1 (0.9)	0 (0.0)	1 (1.1)	
Dyskinesis, *n* (%)	0 (0.0)	0 (0.0)	0 (0.0)	
**Mid segment**				1.000
Normal, *n* (%)	31 (28.2)	5 (29.4)	26 (28.0)	
Hypokinesis, *n* (%)	49 (44.5)	8 (47.1)	41 (44.1)	
Akinesis, *n* (%)	28 (25.5)	4 (23.5)	24 (25.8)	
Dyskinesis, *n* (%)	2 (1.8)	0 (0.0)	2 (2.2)	
**Apical segment**				0.838
Normal, *n* (%)	44 (40.0)	7 (41.2)	37 (39.8)	
Hypokinesis, *n* (%)	31 (28.2)	4 (23.5)	27 (29.0)	
Akinesis, *n* (%)	18 (16.4)	4 (23.5)	14 (15.1)	
Dyskinesis, *n* (%)	17 (15.5)	2 (11.8)	15 (16.1)	
**Tissue mapping**				
**Native T1, ms**				
Global	1102 ± 71	1088 ± 76	1104 ± 71	0.383
Basal	1073 (1036 to 1105)	1064 (1012 to 1111)	1074 (1037 to 1103)	0.367
Mid	1130 ± 86	1126 ± 95	1131 ± 84	0.821
Apical	1120 (1041 to 1208)	1081 (1040 to 1162)	1120 (1044 to 1211)	0.577
**T2, ms**				
Global	56 (53 to 60)	60 (55 to 65)	56 (53 to 59)	0.052
Basal	51 (48 to 54)	52 (49 to 58)	50 (48 to 54)	0.234
Mid	58 (54 to 62)	61 (55 to 69)	57 (53 to 61)	0.068
Apical	61 (55 to 67)	62 (58 to 72)	61 (55 to 66)	0.192
**ECV, %**				
Global	32 (28 to 34)	32 (31 to 34)	31 (28 to 34)	0.355
Basal	29 (26 to 31)	29 (28 to 34)	29 (26 to 31)	0.273
Mid	32 (29 to 35)	33 (31 to 35)	32 (29 to 35)	0.400
Apical	35 ± 6	34 ± 5	35 ± 6	0.405
**LGE**				
No LGE, *n* (%)	83 (75.5)	11 (64.7)	72 (77.4)	0.357
Non-ischaemic LGE, *n* (%)	17 (15.5)	3 (17.6)	14 (15.1)	0.725
Ischaemic LGE, *n* (%)	10 (9.1)	3 (17.6)	7 (7.5)	0.184

CMR, cardiac magnetic resonance imaging; ECV, extracellular volume; EDV, end-diastolic volume; ESV, end-systolic volume; EF, ejection fraction; LGE, late gadolinium enhancement; LV, left ventricular; MACE, major adverse cardiovascular and cerebrovascular events; RV, right ventricular.

### Impact of T2 on MACE

During a median follow-up of 763 days (IQR 525–1357), 17 patients (15.5%) experienced MACE, including death in 6 (5.5%), recurrent TTS in 6 (5.5%), heart failure hospitalization in 3 (2.7%), and stroke/transient ischaemic attack in 2 (1.8%) (see Supplementary data online, *Table S1*). Outcome status was confirmed for all patients at the last contact. Time-dependent ROC at 1 year identified T2 ≥ 63 ms as the optimal threshold (AUC 0.671, 95% CI 0.419–0.921; sensitivity 56%, specificity 89%). Time-dependent AUCs were 0.688 at 2 years (95% CI 0.495–0.881) and 0.674 at 3 years (95% CI 0.512–0.836) (see Supplementary data online, *Table S12* and Supplementary data online, *Figure S7*). Nineteen patients had global T2 ≥ 63 ms and MACE was more frequent compared with the 91 patients with T2 < 63 ms (36.8% vs. 11.0%, *P* = 0.010). Kaplan–Meier curves showed higher MACE rates in patients with myocardial T2 ≥ 63 ms (log-rank *P* = 0.003; *Figure 1*). The prevalence of global T2 above the institutional upper normal (≥55 ms) was 56.4% (62/110) overall and 64.7% (11/17) among MACE-positive patients.

**Figure 1:**
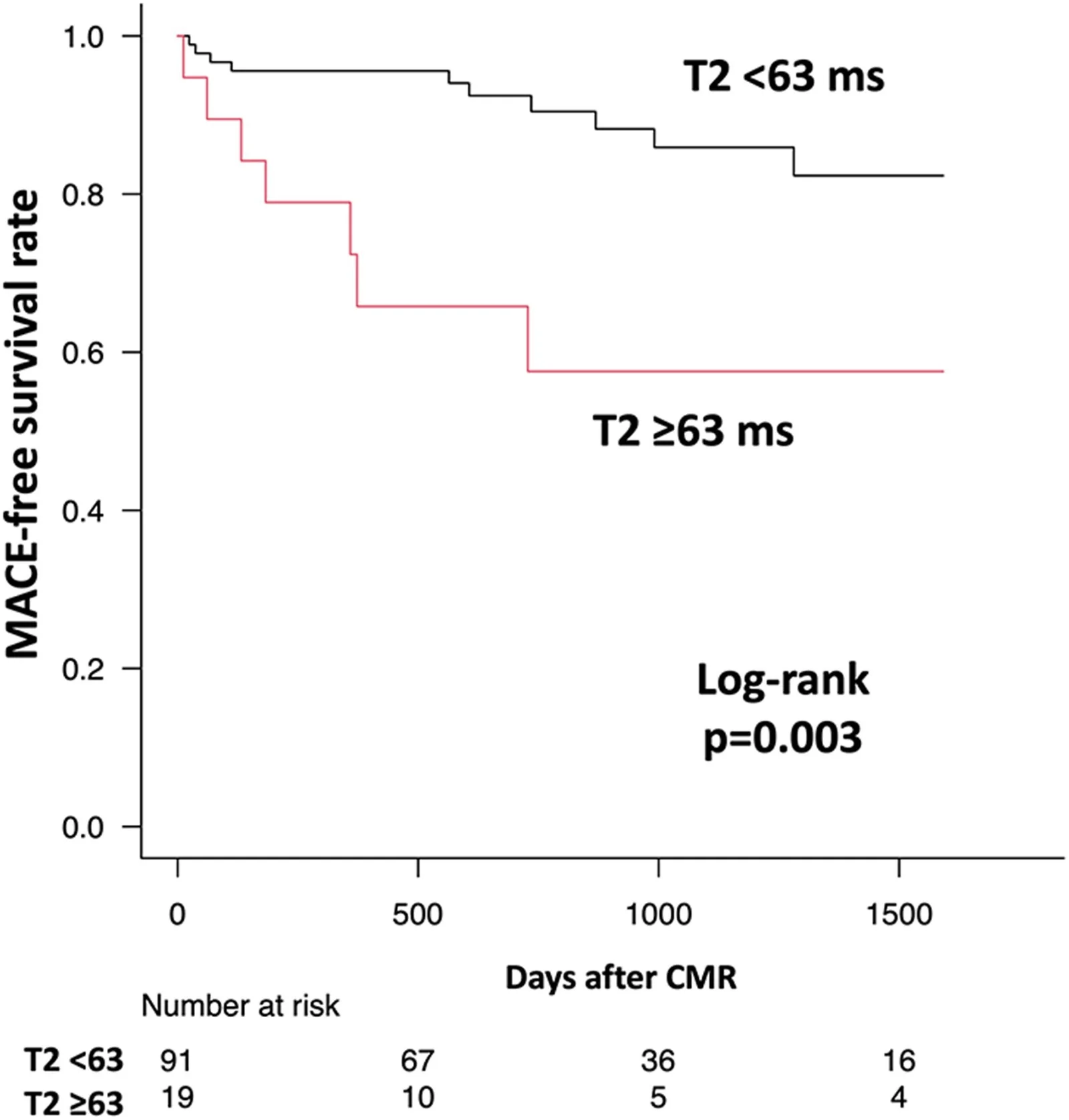
Kaplan–Meier curves for MACE stratified by global T2 ≥ 63 ms vs. <63 ms. MACE, major adverse cardiovascular and cerebrovascular events.

### Multivariable prognostic modelling

The ridge-derived clinical index (based on age, sex, atrial fibrillation, neurologic trigger, obstructive CAD ≥70%, and atypical TTS phenotype) was associated with MACE (HR 5.76 per index unit; 95% CI 2.48–13.36; *P* < 0.001) (*Table 3*). In the clinical + imaging index (adding LVEF, RVEF, ischaemic LGE, and LV mass index to the clinical set), the base model was also associated with MACE (HR 7.80; 95% CI 3.17–19.23; *P* < 0.001). The clinical-only and clinical + imaging models had nearly identical C-indices (0.777 and 0.775, respectively). This small difference likely reflects sampling variability between the separately fitted ridge models; the clinical + imaging model nevertheless had a lower AIC (132.95 vs. 135.41). Adding T2 per 5 ms improved model fit and discrimination (HR 1.40; 95% CI 1.06–1.87; *P* = 0.020; LRT *P* = 0.032; Harrell’s C-index 0.775 to 0.801; AIC 132.95 to 130.35) (*Tables 3–4*, *Figure 2*). Adding T2 ≥ 63 ms also improved fit and achieved the highest apparent discrimination (HR 3.02; 95% CI 1.14–8.00; *P* = 0.026; LRT *P* = 0.035; C-index 0.775 to 0.832; AIC 132.95 to 130.48). The proportional hazards assumption was not violated based on Schoenfeld residuals (global Schoenfeld *P* = 0.102). The bootstrap-corrected 1-year AUC was 0.669 (95% CI 0.388–0.929); the Youden threshold had a tight plausibility range (median 64 ms, IQR 64–66 ms); the bootstrap-corrected C-index of the ridge-adjusted model with continuous T2 was 0.781 (apparent 0.801, optimism 0.020). Calibration was adequate (calibration slope 1.02, calibration-in-the-large 0.00, Brier score 0.056; Supplementary data online, *Figure S5*). Restricted cubic spline analysis did not support a clinically meaningful departure from linearity for T2 (non-linearity *P* = 0.460, AIC of the linear model 1.45 lower than the spline; Supplementary data online, *Figure S6*).

**Figure 2:**
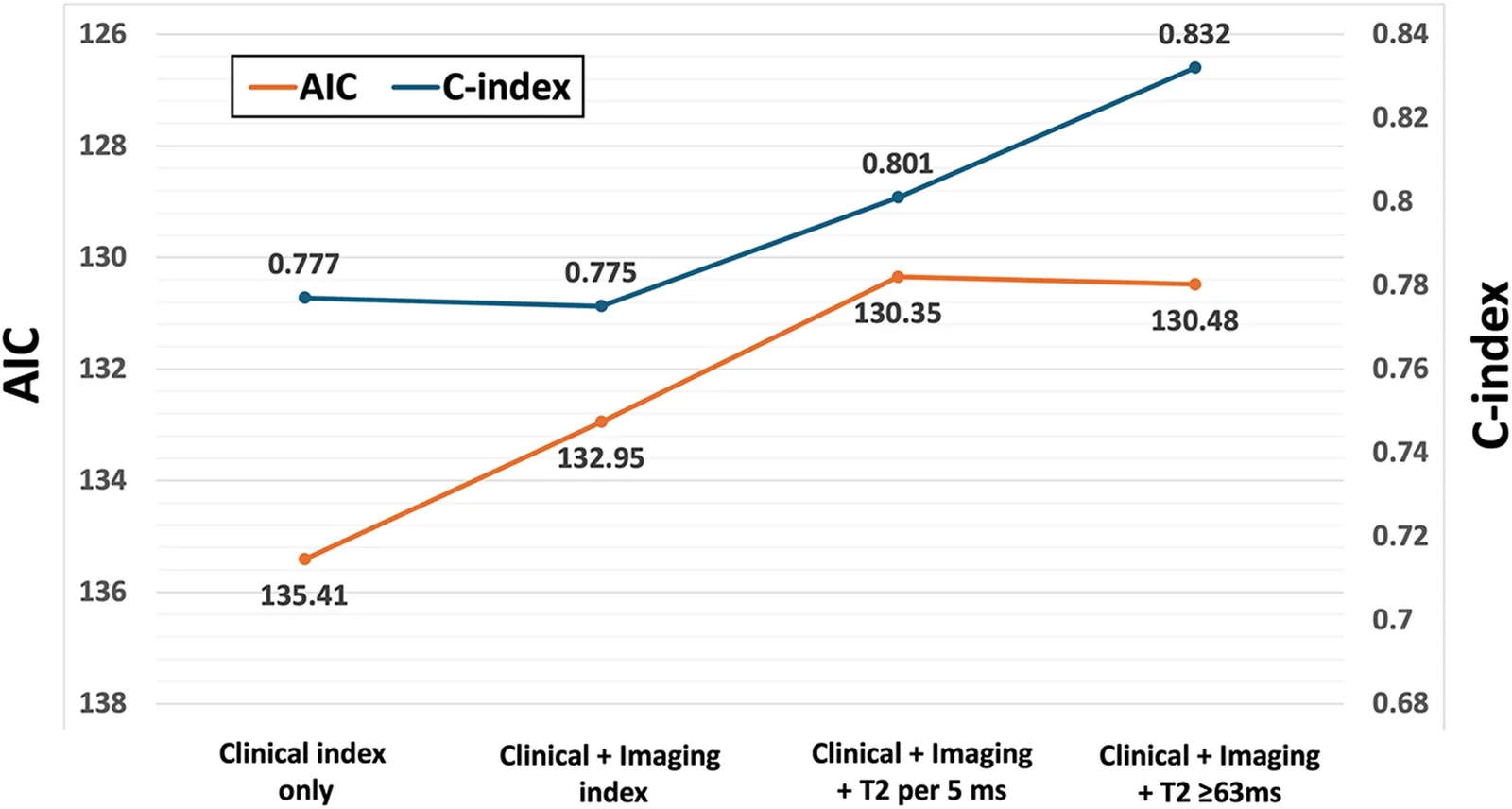
Comparison of model performance using C-index and AIC. AIC, Akaike Information Criterion.

**Table 3:** Ridge-penalized Cox models for predicting MACE: clinical index, clinical + imaging index without myocardial T2, and clinical + imaging index with myocardial T2

Model	Predictor	Hazard Ratio	95% CI	*P*-value
Clinical index only	Ridge clinical index (per unit)	5.76	2.48 to 13.36	<0.001
Clinical + imaging index without T2	Ridge clinical + imaging index (per unit)	7.80	3.17 to 19.23	<0.001
Clinical + imaging index + T2 (per 5 ms)	Ridge clinical + imaging index (per unit)	7.74	2.97 to 20.16	<0.001
Clinical + imaging index + T2 (per 5 ms)	T2 (per 5 ms)	1.40	1.06 to 1.87	0.020
Clinical + imaging index + T2 ≥ 63 ms	Ridge clinical + imaging index (per unit)	7.40	2.85 to 19.21	<0.001
Clinical + imaging index + T2 ≥ 63 ms	T2 ≥ 63 ms	3.02	1.14 to 8.00	0.026

CI, confidence interval; MACE, major adverse cardiovascular and cerebrovascular events.

**Table 4:** Model performance for predicting MACE (global myocardial T2)

Model	C-index (Harrell)	AIC	Likelihood-ratio test
Clinical index only	0.777	135.41	—
Clinical + imaging index without T2	0.775	132.95	—
Clinical + imaging index + T2 (per 5 ms)	0.801	130.35	χ^2^ = 4.61, df = 1, *P* = 0.032 (vs. clinical + imaging index only)
Clinical + imaging index + T2 ≥ 63 ms	0.832	130.48	χ^2^ = 4.47, df = 1, *P* = 0.035 (vs. clinical + imaging index only)

AIC, Akaike Information Criterion; df, degrees of freedom; MACE, major adverse cardiovascular and cerebrovascular events.

In a prespecified sensitivity analysis using Firth-penalized Cox with all covariates entered individually, global T2 remained associated with MACE after adjustment both as a continuous marker (per 5 ms: HR 1.70; 95% CI 1.20–2.40; *P* = 0.004) (*Figure 3* and Supplementary data online, *Table S2*) and as a binary marker (T2 ≥ 63 ms: HR 6.74; 95% CI 1.93–23.87; *P* = 0.003) (*Figure 4* and Supplementary data online, *Table S3*). LV mass index retained an inverse association with MACE risk in both Firth models (per unit: HR 0.93; 95% CI 0.86–0.99; *P* = 0.032 for continuous-T2 model; HR 0.93; 95% CI 0.86–0.999; *P* = 0.045 for binary-T2 model).

**Figure 3:**
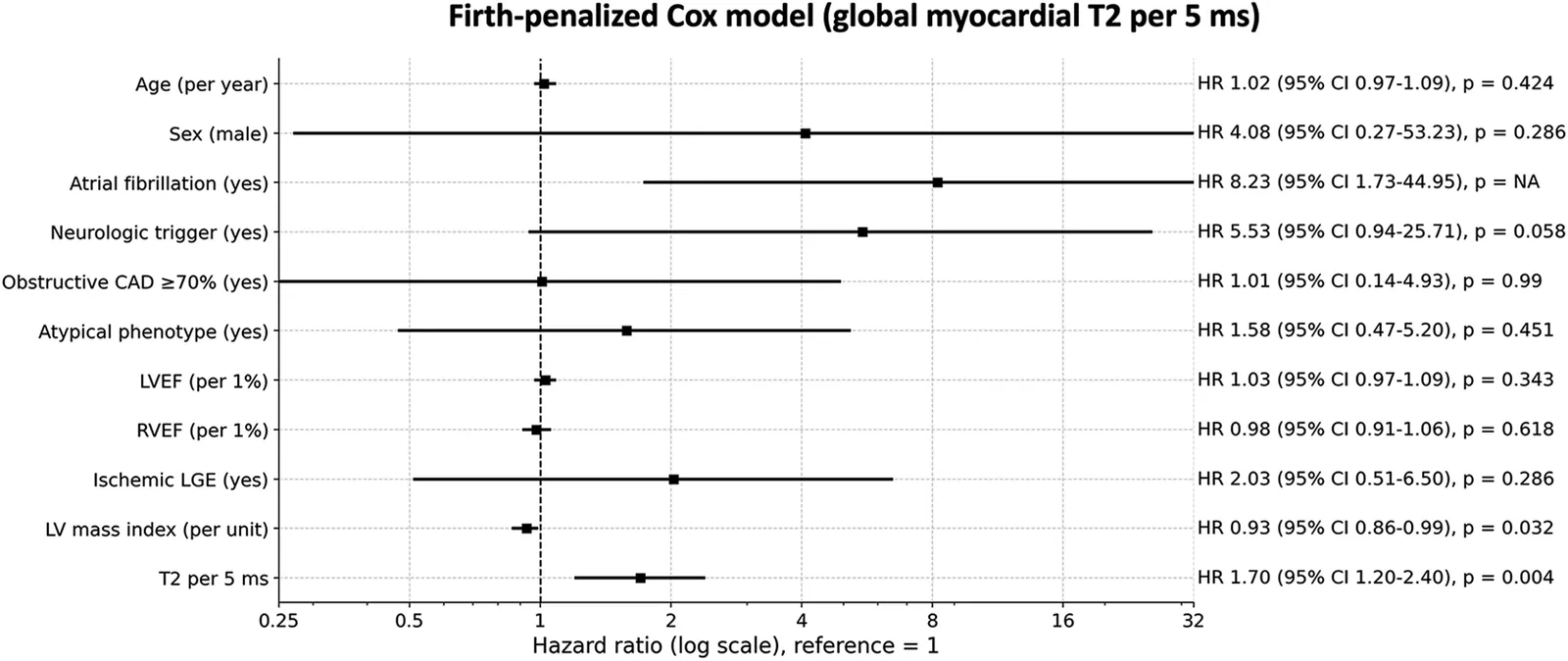
Forest plot for Firth-penalized cox model (global T2 per 5 ms). **‘**NA’ indicates that a *P*-value could not be reliably computed for that variable in the Firth model. Interpretation should be based on the hazard ratio and its 95% CI. CAD, coronary artery disease; CI, confidence interval; EF, ejection fraction; LV, left ventricular; RV, right ventricular; TTS, takotsubo syndrome.

**Figure 4:**
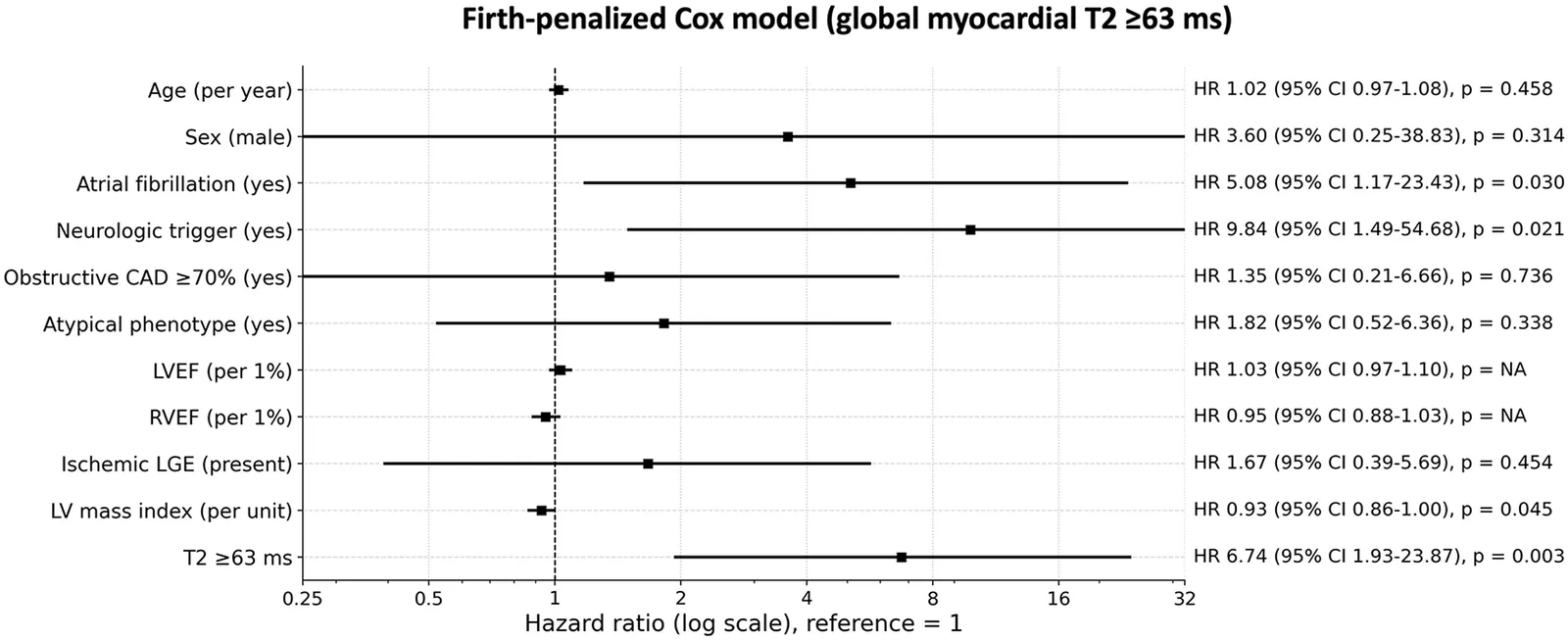
Forest plot for Firth-penalized Cox model (global T2 ≥ 63 ms). **‘**NA’ indicates that a *P*-value could not be reliably computed for that variable in the Firth model. Interpretation should be based on the hazard ratio and its 95% CI. CAD, coronary artery disease; CI, confidence interval; EF, ejection fraction; LV, left ventricular; RV, right ventricular; TTS, takotsubo syndrome.

### Sub-analysis: global native myocardial T1

In analyses substituting global native T1 for T2, continuous T1 (per 100 ms) did not provide incremental prognostic value beyond prespecified clinical and imaging indices (see Supplementary data online, *Tables S4*–*S5*). In the fully adjusted Firth-penalized Cox model, continuous T1 was similarly not independently associated with MACE (HR 0.68; 95% CI 0.29–1.59; *P*-value not available) (see Supplementary data online, *Table S6*). In contrast, native T1 ≥ 1237 ms was associated with a higher risk of MACE (HR 12.66; 95% CI 2.13–58.20; *P* = 0.008); however, only three patients exceeded this threshold, yielding imprecise estimates with wide confidence intervals (see Supplementary data online, *Table S7*).

### Sensitivity analyses

Basal (HR 1.28 per 5 ms, *P* = 0.029), mid (HR 1.26, *P* = 0.026), and region-guided T2 (HR 1.28, *P* = 0.046) were each associated with MACE to a comparable degree, whereas apical T2 alone (HR 1.10, *P* = 0.463) and maximal segmental T2 (the slice with the highest mean T2, irrespective of wall motion; HR 1.22, *P* = 0.057) were not (see Supplementary data online, *Table S8* and Supplementary data online, *Figure S3*). The admission-to-CMR time correlated inversely with global T2 (ρ = −0.21, *P* = 0.028); adjustment for the time interval preserved the T2 effect (HR 1.39) (see Supplementary data online, *Table S9*), and the T2-by-time interval interaction did not converge in the Firth model. In component-specific analyses, the associations with all-cause death and heart failure hospitalization were directionally similar but did not reach statistical significance, whereas the association with recurrent TTS was statistically significant. The stroke/TIA estimate was not interpretable owing to the small event count and complete separation (see Supplementary data online, *Table S10*). The T2–MACE association was further preserved after adjustment for admission TTE and the LVEF-recovery indicator (*n* = 106; HR 1.60), for in-hospital beta-blocker, ACE-I/ARB, diuretic, and inotropic therapy (*n* = 110; HR 1.50), and after exclusion of the 10 patients with ischaemic LGE (HR 1.51) (see Supplementary data online, *Tables S11*, *S13*, *S14*). At the segment level, T2 also scaled with wall-motion severity (mean T2 54.1, 60.4, 63.3, and 65.2 ms in normokinetic, hypokinetic, akinetic, and dyskinetic segments, respectively, *P* < 0.001; Supplementary data online, *Table S15*).

## Discussion

Our results demonstrated that CMR-derived myocardial T2 provided incremental prognostic information beyond prespecified clinical and imaging indices in patients with TTS. Adding continuous global myocardial T2 improved model fit and discrimination, and T2 remained independently associated with MACE in a fully adjusted Firth-penalized Cox model. The internally derived ≥63 ms threshold yielded the highest apparent C-index, but only 19 patients exceeded it, warranting cautious interpretation. Collectively, these results support the potential role for CMR-derived T2 mapping in risk stratification of patients with TTS.

To our knowledge, this is the first study to directly link global T2 value with clinical outcomes in TTS. Previous studies demonstrated that T2 values are significantly elevated in TTS patients compared with controls.^19–21^ More recently, it was reported that increasing T2 values correlated inversely with LV systolic function^12–13^ and were linked to overall disease severity, as measured by the GEIST risk score.^12^ Although these associations suggested the T2 value as a potential prognostic marker, the direct relationship between T2 and clinical outcomes has never been assessed. Our results extend prior literature by linking higher global T2 to subsequent MACE, suggesting that the extent of acute myocardial oedema carries incremental prognostic information beyond clinical indices and conventional ventricular function measures.

Although native T1 and ECV are frequently elevated in TTS,^12,18–20^ both are less specific to myocardial oedema and can reflect other disease processes, including fibrosis or infiltration.^22^ In contrast, T2 mapping quantifies free-water content and, is, therefore a more direct readout of myocardial oedema, the pathophysiologic hallmark of acute TTS. Consistent with this, prior work showed that the regional T2 dispersion (apicobasal gradient) correlates with electrocardiographic repolarization abnormalities, whereas neither T1 nor ECV demonstrated this association.^12^ In our cohort, native myocardial T1 was not consistently associated with MACE in the sub-analysis (see Supplementary data online, materials). In contrast, T2 remained a more consistent and robust risk predictor across modelling strategies. Taken together, these considerations suggest that T2 is a more specific marker of acute disease burden in TTS than T1 or ECV.

Regional analyses provided a better understanding of the global T2 signal. In the same ridge-adjusted model, basal, mid, and region-guided T2 each showed associations of comparable magnitude with MACE, whereas apical T2 alone did not, and global T2 retained the strongest association (HR 1.40 per 5 ms; *Table 3*). Two considerations may explain our finding. The TTS cohort is phenotypically heterogeneous, with 32.7% of patients showing atypical variants (mid-ventricular 24.5%, reverse 3.6%, focal 4.5%) in which the regional injury is not centred at the apex. An apically anchored exposure therefore misclassifies the affected territory in these patients. Additionally, apical partial-volume averaging may affect the accuracy of measurements. Global T2 integrates regional variability in wall motion and myocardial oedema across the entire LV, supporting its retention as the primary exposure.

A methodological strength of our study is the use of ridge-penalized Cox models to create a single linear predictor for adjustment, reducing overfitting and avoiding data-driven selection. The negligible difference in C-index between the clinical-only and clinical + imaging models likely reflects sampling variability in this low-event cohort. We then evaluated the incremental value of T2, a strategy that prioritizes parsimony and reproducibility and aligns with contemporary guidance for prognostic modelling in modest samples.^23^ As a robustness check, Firth-penalized analyses, which mitigate small-sample bias, yielded concordant results for T2. Among covariates, atrial fibrillation and neurologic triggers were associated with higher risk in the binary-T2 model (see Supplementary data online, *Table S3* and *Figure 4*), consistent with prior reports.^3,24^ The inverse association of LV mass index is a novel finding. In our cohort, 36.4% of patients had low LV mass, defined as <2 SD below reference values,^25^ which may reflect generalized deconditioning, cachexia, or cardio-sarcopenia.^26,27^ In such patients, low LV mass may indicate a higher-risk disease state, whereas patients with normal LV mass are seemingly relatively protected. Further prospective studies are needed to test this hypothesis.

Given the non-benign course of TTS, reliable risk stratification is essential. Current recommendations emphasize CMR primarily as an adjunctive test to facilitate the diagnosis of TTS.^17^ Our data support adding myocardial T2 mapping to routine TTS protocols not only for diagnosis but also for risk assessment. In centres with mapping capability, reporting global T2 may help identify patients who warrant closer follow-up after discharge. While our study identified T2 ≥ 63 ms as a high-risk threshold, this finding was not externally validated and therefore should not be adopted into clinical practice. In addition, because improvements in discrimination were modest, T2 should complement rather than replace clinical judgement and established risk markers.

### Study limitations

This study has several limitations. The retrospective, single-centre design and limited number of events (*n* = 17) constrain precision: a fully multivariable analysis without penalization was not feasible, confidence intervals for several covariates are correspondingly wide, and penalized estimates are conservative by construction. We mitigated overfitting through penalization and confirmed proportional hazards assumptions. We acknowledge, however, that ridge penalization introduces shrinkage towards zero, making the reported HRs conservative estimates of the unpenalized effect, and that the cross-validated lambda is itself subject to sampling variability in low-event data. Ridge-penalised Cox is therefore a principled accommodation to a small-event setting rather than a substitute for external validation in larger, multi-centre cohorts with standardized mapping protocols and coordinated imaging timing. CMR timing varied within our cohort, and it is possible that earlier scans would result in higher T2 values and a greater association with outcome. The timing-stratified analyses, while reassuring, were not powered to demonstrate equivalence across the full window. NT-proBNP was not measured systematically and was therefore not included. CMR feature-tracking strain was not part of the clinical reporting workflow and was not systematically quantified. The myocardial T2 cut-off of 63 ms is internally derived and exploratory, and thus requires external validation before clinical adoption. Finally, downstream markers that may lie on the causal pathway between oedema and outcome (e.g. QTc prolongation) were not included in the primary models to avoid over-adjustment for potential mediators.

## Conclusion

Quantitative myocardial T2 may provide incremental prognostic information in TTS beyond ridge-derived clinical and imaging indices. Incorporating T2 into diagnostic CMR protocols may refine risk stratification. These results warrant external validation in larger, multi-centre cohorts with standardized mapping and timing.

## Supplementary Material

jeag243_Supplementary_Data

## Data Availability

The authors are willing to share study data upon reasonable request and according to institutional regulations.
