# Supplementary material for: Prognostic value of cardiac magnetic resonance global T2 mapping in patients with takotsubo syndrome
Source: Eur Heart J Cardiovasc Imaging. 2026 Sep 7;27(11):1955–66. doi: 10.1093/ehjci/jeag243 (PMC13721763; doi:10.1093/ehjci/jeag243)
Supplement: Supplementary file 1 [file jeag243_supplementary_data.docx]

**Supplemental Materials**

**Supplemental Figures**

**Figure S1. Patient flow chart**

CMR, cardiac magnetic resonance imaging; LGE, late gadolinium enhancement; TTS, Takotsubo syndrome

**Figure S2. Representative CMR images in Takotsubo syndrome with elevated global myocardial T2 value and subsequent death**

A 76-year-old woman presented with chest pain and was diagnosed with Takotsubo syndrome. Transthoracic echocardiography demonstrated apical ballooning with severe LV systolic dysfunction (LVEF 18%). Repeat echocardiography at day 5 showed improvement in LV function (LVEF 43%), and she was discharged without in-hospital complications. On index CMR, global myocardial T2 was 64 ms. Six months later, she was hospitalised for pneumonia and died after pneumonia complicated by type 2 myocardial infarction.

A-B: Balanced steady-state free precession three-chamber cine images showing apical ballooning (arrows) (A: systole, B: diastole)

C-D: Myocardial T1 mapping with global native T1 value of 1246 ms (institutional reference: normal <1040 ms). (C: three-chamber view, D: polar map with segmental native T1 values)

E-F: Myocardial T2 mapping with global T2 value of 64 ms (institutional reference: normal <55 ms). (E: three-chamber view, F: polar map with segmental T2 values)

CMR, cardiac magnetic resonance; EF, ejection fraction; LV, left ventricular

**Figure S3.** **Forest plot of T2 parametrisations**

Hazard ratios (per 5 ms increase) for MACE from separate ridge-adjusted Cox proportional-hazards models comparing six T2 parametrisations: global, region-guided, maximal segmental, apical, mid, and basal. Region-guided T2 was defined as the mean T2 of the slice (or slices, in the case of ties) exhibiting the worst wall-motion grade per patient. Maximal segmental T2 was defined as the slice (basal, mid-ventricular, or apical) with the highest mean T2, irrespective of wall-motion abnormality. Solid squares are point estimates and horizontal lines are 95% confidence intervals; the vertical dashed line is the null reference (HR = 1). The Global T2 row reproduces the primary analysis estimate from Table 3. Numerical values to the right of each bar are HR, 95% CI, and Wald p-value.

CI, confidence interval; HR, hazard ratio; MACE, major adverse cardiovascular and cerebrovascular events

**Figure S4. Admission-to-CMR interval and its relationship with global T2**

(A) Histogram of the admission-to-CMR interval (n = 110); the vertical dashed line marks the cohort median (4 days). (B) Scatter of admission-to-CMR interval against global T2, with patients coloured by MACE status; the black curve is a LOESS smoother (span = 0.9) with its 95% pointwise confidence band. The Spearman correlation between interval and global T2 was rho = -0.21 (p=0.028), consistent with progressive resolution of myocardial oedema.

CMR, cardiac magnetic resonance; LOESS, locally weighted scatterplot smoothing; MACE, major adverse cardiovascular and cerebrovascular events.

**Figure S5. One-year calibration plot of the ridge-adjusted Cox model**

Model-predicted versus observed 1-year MACE probability for the ridge-adjusted Cox model with continuous global T2; the dashed 45-degree line denotes perfect calibration. The dotted grey line (cross markers) is the apparent calibration and the solid blue line (filled circles) is the bootstrap-corrected calibration after 200 resamples. Both lie close to the diagonal across the observed range of predicted probabilities. Calibration slope = 1.02 (ideal = 1.0), calibration-in-the-large = 0.00 (ideal = 0), Brier score = 0.056.

MACE, major adverse cardiovascular and cerebrovascular events

**Figure S6. Restricted cubic spline of MACE risk against global T2**

Hazard ratio for MACE on the log scale (y-axis) as a function of global T2 (x-axis), modelled with a 3-knot restricted cubic spline (RCS) within the ridge-adjusted Cox framework and referenced at 55 ms (institutional upper-normal limit; black dotted line). The solid blue curve is the RCS fit and the shaded band is its 95% pointwise confidence interval. The orange dotted line marks the Youden-derived threshold of 63 ms. The relationship is approximately log-linear (likelihood-ratio test for nonlinearity p=0.460; AIC of the linear model 1.45 units lower than that of the RCS model), supporting the linear "per 5 ms" coding used in the primary analysis.

AIC, Akaike Information Criterion; MACE, major adverse cardiovascular and cerebrovascular events; RCS, restricted cubic spline

**Figure S7. Time-dependent ROC curves for global T2 at 1, 2, and 3 years**

Sensitivity versus 1 - specificity for global T2 as a continuous prognostic marker for MACE at the three follow-up horizons. The dashed 45-degree line denotes the line of no discrimination (AUC = 0.5). AUCs were 0.671 (95% CI 0.419–0.921) at 1 year, 0.688 (95% CI 0.495–0.881) at 2 years, and 0.674 (95% CI 0.512–0.836) at 3 years, indicating preserved discrimination beyond the early post-presentation period.

AUC, area under the curve; CI, confidence interval; MACE, major adverse cardiovascular and cerebrovascular events; ROC, receiver operating characteristic

**Supplemental Tables**

**Table S1. Summary of endpoints**

| **Parameters** | **Total**  **(n=110)** | **T2 ≥63 ms**  **(n=19)** | **T2 <63 ms**  **(n=91)** | **p-value** |
| --- | --- | --- | --- | --- |
| **Primary endpoint** |  |  |  |  |
| MACE, *n* (%) | 17 (15.5) | 7 (36.8) | 10 (11.0) | 0.010 |
| **Individual event** |  |  |  |  |
| All-cause death, *n* (%) | 6 (5.5) | 2 (10.5) | 4 (4.4) | 0.276 |
| Recurrent TTS, *n* (%) | 6 (5.5) | 3 (15.8) | 3 (3.3) | 0.063 |
| Heart failure hospitalisation, *n* (%) | 3 (2.7) | 2 (10.5) | 1 (1.1) | 0.077 |
| Stroke or TIA, *n* (%) | 2 (1.8) | 0 (0.0) | 2 (2.2) | 1.000 |
| **Follow-up period, days** | 763 (525 to 1357) | 632 (371 to 1205) | 784 (534 to 1555) | 0.393 |

Categorical variables are presented as number (percentage).

Group comparisons used Pearson χ² or Fisher exact tests for categorical variables and Student t test or Mann–Whitney U test for continuous variables, as appropriate.

MACE, major adverse cardiovascular and cerebrovascular events; TIA, transient ischaemic attack; TTS, Takotsubo syndrome

**Table S2. Firth-penalised Cox model (global myocardial T2 per 5 ms)**

| **Covariate** | **Hazard Ratio** | **95% CI** | **p-value** |
| --- | --- | --- | --- |
| Age (per year) | 1.02 | 0.97 to 1.09 | 0.424 |
| Sex (male) | 4.08 | 0.27 to 53.23 | 0.286 |
| Atrial fibrillation (yes) | 8.23 | 1.73 to 44.95 | NA |
| Neurologic trigger (yes) | 5.53 | 0.94 to 25.71 | 0.058 |
| Obstructive CAD ≥70% (yes) | 1.01 | 0.14 to 4.93 | 0.990 |
| Atypical phenotype (yes) | 1.58 | 0.47 to 5.20 | 0.451 |
| LVEF (per 1%) | 1.03 | 0.97 to 1.09 | 0.343 |
| RVEF (per 1%) | 0.98 | 0.91 to 1.06 | 0.618 |
| Ischaemic LGE (present) | 2.03 | 0.51 to 6.50 | 0.286 |
| LV mass index (per unit) | 0.93 | 0.86 to 0.99 | 0.032 |
| T2 (per 5 ms) | 1.70 | 1.20 to 2.40 | 0.004 |

“NA” indicates that a p-value could not be reliably computed for that variable in the Firth model. Interpretation should be based on the hazard ratio and its 95% CI.

CAD, coronary artery disease; CI, confidence interval; EF, ejection fraction; LGE, late gadolinium enhancement; LV, left ventricular; RV, right ventricular

**Table S3. Firth-penalised Cox model (global myocardial T2 ≥63 ms)**

| **Covariate** | **Hazard Ratio** | **95% CI** | **p-value** |
| --- | --- | --- | --- |
| Age (per year) | 1.02 | 0.97 to 1.08 | 0.458 |
| Sex (male) | 3.60 | 0.25 to 38.83 | 0.314 |
| Atrial fibrillation (yes) | 5.08 | 1.17 to 23.43 | 0.030 |
| Neurologic trigger (yes) | 9.84 | 1.49 to 54.68 | 0.021 |
| Obstructive CAD ≥70% (yes) | 1.35 | 0.21 to 6.66 | 0.736 |
| Atypical phenotype (yes) | 1.82 | 0.52 to 6.36 | 0.338 |
| LVEF (per 1%) | 1.03 | 0.97 to 1.10 | NA |
| RVEF (per 1%) | 0.95 | 0.88 to 1.03 | NA |
| Ischaemic LGE (present) | 1.67 | 0.39 to 5.69 | 0.454 |
| LV mass index (per unit) | 0.93 | 0.86 to 0.999 | 0.045 |
| T2 ≥63 ms | 6.74 | 1.93 to 23.87 | 0.003 |

“NA” indicates that a p-value could not be reliably computed for that variable in the Firth model. Interpretation should be based on the hazard ratio and its 95% CI.

CAD, coronary artery disease; CI, confidence interval; EF, ejection fraction; LGE, late gadolinium enhancement; LV, left ventricular; RV, right ventricular

**Table S4. Ridge-penalised Cox models for predicting MACE: clinical index, clinical + imaging index without myocardial T1, and clinical + imaging index with myocardial T1**

| **Model** | **Predictor** | **Hazard Ratio** | **95% CI** | **p-value** |
| --- | --- | --- | --- | --- |
| Clinical index only | Ridge clinical index (per unit) | 5.76 | 2.48 to 13.36 | <0.001 |
| Clinical + imaging index without T1 | Ridge clinical + imaging index (per unit) | 7.80 | 3.17 to 19.23 | <0.001 |
| Clinical + imaging index + T1 (per 100 ms) | Ridge clinical + imaging index (per unit) | 7.54 | 3.06 to 18.59 | <0.001 |
| Clinical + imaging index + T1 (per 100 ms) | T1 (per 100 ms) | 0.67 | 0.34 to 1.33 | 0.253 |
| Clinical + imaging index + T1 ≥1237 ms | Ridge clinical + imaging index (per unit) | 9.27 | 3.63 to 23.67 | <0.001 |
| Clinical + imaging index + T1 ≥1237 ms | T1 ≥1237 ms | 9.89 | 2.10 to 46.57 | 0.004 |

Ridge indices were built by 10-fold cross-validated ridge Cox using prespecified covariates. Sample size n=110, events=17. The clinical index included age, sex, atrial fibrillation, neurologic trigger, obstructive coronary artery disease ≥70%, and atypical TTS phenotype. The clinical + imaging index added LVEF, RVEF, ischaemic LGE, and LV mass index.

CI, confidence interval; EF, ejection fraction; LGE, late gadolinium enhancement; LV, left ventricular; MACE, major adverse cardiovascular and cerebrovascular events; RV, right ventricular; TTS, Takotsubo syndrome

**Table S5. Model performance for predicting MACE (global myocardial native T1)**

| **Model** | **C-index (Harrell)** | **AIC** | **Likelihood-ratio test** |
| --- | --- | --- | --- |
| Clinical index only | 0.777 | 135.41 | — |
| Clinical + imaging index without T1 | 0.775 | 132.95 | — |
| Clinical + imaging index + T1 (per 100 ms) | 0.758 | 133.62 | χ²=1.33, df=1, p=0.249 (vs clinical + imaging index only) |
| Clinical + imaging index + T1 ≥1237 ms | 0.828 | 129.76 | χ²=5.20, df=1, p=0.023 (vs clinical + imaging index only) |

C-index is Harrell’s C computed from Cox models. Likelihood-ratio tests compare each model with T1 to the corresponding model without T1. Sample size n=110, events=17. The clinical index included age, sex, atrial fibrillation, neurologic trigger, obstructive coronary artery disease ≥70%, and atypical TTS phenotype. The clinical + imaging index added LVEF, RVEF, ischaemic LGE, and LV mass index.

AIC, Akaike Information Criterion; df, degrees of freedom; EF, ejection fraction; LGE, late gadolinium enhancement; LV, left ventricular; MACE, major adverse cardiovascular and cerebrovascular events; RV, right ventricular

**Table S6. Firth-penalised Cox model (global myocardial native T1 per 100ms)**

| **Covariate** | **Hazard Ratio** | **95% CI** | **p-value** |
| --- | --- | --- | --- |
| Age (per year) | 1.04 | 0.98 to 1.10 | 0.205 |
| Sex (male) | 1.87 | 0.12 to 20.36 | 0.621 |
| Atrial fibrillation (yes) | 5.95 | 1.37 to 26.58 | 0.018 |
| Neurologic trigger (yes) | 3.86 | 0.67 to 16.49 | 0.117 |
| Obstructive CAD ≥70% (yes) | 1.26 | 0.25 to 4.92 | 0.764 |
| Atypical phenotype (yes) | 1.47 | 0.44 to 4.56 | 0.515 |
| LVEF (per 1%) | 1.00 | 0.95 to 1.06 | 0.951 |
| RVEF (per 1%) | 0.99 | 0.91 to 1.05 | 0.673 |
| Ischaemic LGE (present) | 1.05 | 0.24 to 3.46 | 0.939 |
| LV mass index (per unit) | 0.97 | 0.90 to 1.04 | 0.365 |
| T1 (per 100 ms) | 0.68 | 0.29 to 1.59 | NA |

“NA” indicates that a p-value could not be reliably computed for that variable in the Firth model. Interpretation should be based on the hazard ratio and its 95% CI.

CAD, coronary artery disease; CI, confidence interval; EF, ejection fraction; LGE, late gadolinium enhancement; LV, left ventricular; RV, right ventricular

**Table S7. Firth-penalised Cox model (global myocardial native T1 ≥1237 ms)**

| **Covariate** | **Hazard Ratio** | **95% CI** | **p-value** |
| --- | --- | --- | --- |
| Age (per year) | 1.03 | 0.98 to 1.10 | 0.281 |
| Sex (male) | 3.43 | 0.19 to 42.55 | 0.366 |
| Atrial fibrillation (yes) | 7.71 | 1.62 to 38.44 | 0.011 |
| Neurologic trigger (yes) | 4.40 | 0.75 to 20.37 | 0.094 |
| Obstructive CAD ≥70% (yes) | 1.24 | 0.24 to 5.21 | 0.783 |
| Atypical phenotype (yes) | 1.07 | 0.31 to 3.39 | 0.912 |
| LVEF (per 1%) | 1.02 | 0.96 to 1.08 | 0.508 |
| RVEF (per 1%) | 0.97 | 0.89 to 1.04 | 0.438 |
| Ischaemic LGE (present) | 1.10 | 0.22 to 3.95 | 0.893 |
| LV mass index (per unit) | 0.94 | 0.88 to 1.01 | 0.095 |
| T1 ≥1237 ms | 12.66 | 2.13 to 58.20 | 0.008 |

CAD, coronary artery disease; CI, confidence interval; EF, ejection fraction; LGE, late gadolinium enhancement; LV, left ventricular; RV, right ventricular

| **T2 parametrisation** | **Hazard Ratio per 5 ms increase in T2** | **95% CI** | **p-value** |
| --- | --- | --- | --- |
| Global T2 | 1.40 | 1.06 to 1.87 | 0.020 |
| Region-guided T2 (based on worst WMA) | 1.28 | 1.00 to 1.62 | 0.046 |
| Maximal segmental T2 | 1.22 | 0.99 to 1.49 | 0.057 |
| Apical T2 | 1.10 | 0.85 to 1.42 | 0.463 |
| Mid T2 | 1.26 | 1.03 to 1.54 | 0.026 |
| Basal T2 | 1.28 | 1.03 to 1.59 | 0.029 |

**Table S8. Segmental and region-guided T2: ridge-adjusted Cox models**
Each row represents a separate ridge-adjusted Cox model (standard partial-likelihood) in which the indicated T2 parametrisation was entered together with the ridge-derived clinical-plus-imaging linear predictor.

CI, confidence interval; WMA, wall-motion abnormality

| **Analysis** | **n** | **Hazard Ratio per 5 ms increase in T2** | **95% CI** | **p-value** |
| --- | --- | --- | --- | --- |
| Spearman correlation (interval vs T2) | 110 | rho = -0.21 | - | 0.028 |
| Adjusted for admission-to-CMR interval | 110 | 1.39 | 1.04 to 1.86 | 0.025 |
| ≤4 days | 62 | 1.70 | 0.95 to 3.07 | 0.077 |
| >4 days | 48 | 1.37 | 0.96 to 1.97 | 0.087 |
| T2 x interval interaction | 110 | NA | NA | NA |

**Table S9. Sensitivity analyses anchored on the admission-to-CMR interval**
All Cox models are ridge-adjusted standard Cox regressions with global T2 (per 5 ms) as the exposure. NA = not available (the Firth interaction model did not converge owing to the small number of late-imaging events). The first row shows the unadjusted Spearman correlation between admission-to-CMR interval and global T2.

CI, confidence interval; CMR, cardiac magnetic resonance

| **Component** | **Events (n)** | **Hazard Ratio per 5 ms increase in T2** | **95% CI** | **p-value** |
| --- | --- | --- | --- | --- |
| All-cause death | 6 | 1.50 | 0.95 to 2.38 | 0.078 |
| Heart failure hospitalisation | 3 | 1.45 | 0.83 to 2.53 | 0.190 |
| Recurrent TTS | 6 | 1.85 | 1.20 to 2.86 | 0.005 |
| Stroke or TIA | 2 | NA | NA | NA |

**Table S10. Per-component analysis of the composite endpoint**

Each row represents a separate ridge-adjusted Cox model (standard partial-likelihood; Firth penalisation used as a fallback for small-event components) with the indicated component as the outcome and global T2 (per 5 ms) as the exposure. NA = not available (the estimate for stroke or TIA was not interpretable owing to the small event count [n = 2] and complete separation in the penalised model, which produced a degenerate boundary estimate).

CI, confidence interval; TIA, transient ischaemic attack; TTS, Takotsubo syndrome.

| **Cohort** | **n** | **Hazard Ratio per 5 ms increase in T2** | **95% CI** | **p-value** |
| --- | --- | --- | --- | --- |
| Primary analysis (full cohort) | 110 | 1.40 | 1.06 to 1.87 | 0.020 |
| Excluding ischaemic LGE | 100 | 1.51 | 1.10 to 2.07 | 0.010 |

**Table S11. Sensitivity analysis excluding patients with ischaemic LGE**

Both Cox models are ridge-adjusted standard Cox regressions with global T2 (per 5 ms). Patients with ischaemic LGE (n = 10, 9.1%) were excluded for the sensitivity analysis. The primary analysis row reproduces the primary ridge-adjusted estimate from Table 3 for direct comparison.

CI, confidence interval; LGE, late gadolinium enhancement

| **Time horizon** | **AUC** | **95% CI** |
| --- | --- | --- |
| 1 year | 0.671 | 0.419 to 0.921 |
| 2 years | 0.688 | 0.495 to 0.881 |
| 3 years | 0.674 | 0.512 to 0.836 |

**Table S12. Time-dependent area under the receiver operating characteristic curve (AUC) for global T2**

Time-dependent AUCs were estimated using the inverse-probability-of-censoring-weighted estimator (timeROC R package, marginal weighting). Confidence intervals are reported on the natural scale.

AUC, area under the receiver operating characteristic curve; CI, confidence interval

| **Analysis** | **n** | **Hazard Ratio per 5 ms increase in T2** | **95% CI** | **p-value** |
| --- | --- | --- | --- | --- |
| Primary analysis (ridge-adjusted) | 110 | 1.40 | 1.06 to 1.87 | 0.020 |
| Echo-adjusted Cox model (subset) | 106 | 1.60 | 1.16 to 2.22 | 0.005 |

**Table S13. Sensitivity analysis adjusted for baseline echocardiographic parameters at admission and LVEF recovery**
Adjustment set in the echo-adjusted model: admission LVEF, LVOT obstruction (peak gradient ≥30 mmHg), moderate-or-greater MR, RV dysfunction, LVEF recovery at follow-up TTE/CMR, and the ridge-derived clinical-plus-imaging linear predictor. Four patients (3.6%) had non-retrievable baseline TTE data and were excluded only from this sensitivity analysis.

CI, confidence interval; CMR, cardiac magnetic resonance; LVEF, left ventricular ejection fraction; LVOT, left ventricular outflow tract; MR, mitral regurgitation; RV, right ventricular; TTE, transthoracic echocardiography

| **Analysis** | **n** | **Hazard Ratio per 5 ms increase in T2** | **95% CI** | **p-value** |
| --- | --- | --- | --- | --- |
| Primary analysis (ridge-adjusted) | 110 | 1.40 | 1.06 to 1.87 | 0.020 |
| In-hospital-therapy-adjusted Cox model | 110 | 1.50 | 1.12 to 2.02 | 0.007 |

**Table S14. Sensitivity analysis adjusted for in-hospital medical therapy**
Adjustment set in the therapy-adjusted model: in-hospital therapy of beta-blocker, ACE-I/ARB, diuretics and inotropic support, plus the ridge-derived clinical-plus-imaging linear predictor. Mechanical circulatory support was not used in any patient (0/110) and was therefore excluded from the adjustment formula. Frequencies of in-hospital therapies were: beta-blocker 84/110 (76.4%), ACE-I/ARB 95/110 (86.4%), diuretics 21/110 (19.1%), inotropic support 15/110 (13.6%)

ACE-I, angiotensin-converting enzyme inhibitor; ARB, angiotensin receptor blockers; CI, confidence interval

## **Table S15. T2 by wall-motion severity (segment-level analysis; 330 segments from 110 patients)**

| Wall-motion category | n | Mean (SD) T2, ms |
| --- | --- | --- |
| Normal | 172 | 54.1 (8.1) |
| Hypokinesis | 92 | 60.4 (9.9) |
| Akinesis | 47 | 63.3 (8.6) |
| Dyskinesis | 19 | 65.2 (7.3) |

P-values are from a linear mixed-effects model with a patient-level random intercept; overall p<0.001. Compared with hypokinetic segments, T2 was higher in akinetic (+3.1 ms, p=0.028) and dyskinetic (+5.0 ms, p=0.008) segments.

SD, standard deviation
